# Evasion of tumours from the control of the immune system: consequences of brief encounters

**DOI:** 10.1186/1745-6150-7-31

**Published:** 2012-09-25

**Authors:** Mohannad Al-Tameemi, Mark Chaplain, Alberto d’Onofrio

**Affiliations:** 1 Division of Mathematics, University of Dundee, Dundee, Scotland, UK; 2 Department of Experimental Oncology, European Institute of Oncology, , Via Ripamonti 435, Milano, I-20141, Italy

**Keywords:** Tumour growth, Immune response, Cytotoxic T-lymphocytes, Immuno-evasion, Mathematical models, Chemotaxis, Diffusion, Immuno-editing

## Abstract

**Background:**

In this work a mathematical model describing the growth of a solid tumour in the presence of an immune system response is presented. Specifically, attention is focused on the interactions between cytotoxic T-lymphocytes (CTLs) and tumour cells in a small, avascular multicellular tumour. At this stage of the disease the CTLs and the tumour cells are considered to be in a state of dynamic equilibrium or cancer dormancy. The precise biochemical and cellular mechanisms by which CTLs can control a cancer and keep it in a dormant state are still not completely understood from a biological and immunological point of view. The mathematical model focuses on the spatio-temporal dynamics of tumour cells, immune cells, chemokines and “chemorepellents” in an immunogenic tumour. The CTLs and tumour cells are assumed to migrate and interact with each other in such a way that lymphocyte-tumour cell complexes are formed. These complexes result in either the death of the tumour cells (the normal situation) or the inactivation of the lymphocytes and consequently the survival of the tumour cells. In the latter case, we assume that each tumour cell that survives its “brief encounter” with the CTLs undergoes certain beneficial phenotypic changes.

**Results:**

We explore the dynamics of the model under these assumptions and show that the process of immuno-evasion can arise as a consequence of these encounters. We show that the proposed mechanism not only shape the dynamics of the total number of tumor cells and of CTLs, but also the dynamics of their spatial distribution. We also briefly discuss the evolutionary features of our model, by framing them in the recent quasi-Lamarckian theories.

**Conclusions:**

Our findings might have some interesting implication of interest for clinical practice. Indeed, immuno-editing process can be seen as an “involuntary” antagonistic process acting against immunotherapies, which aim at maintaining a tumor in a dormant state, or at suppressing it.

**Reviewers:**

This article was reviewed by G. Bocharov (nominated by V. Kuznetsov, member of the Editorial Board of *Biology Direct*), M. Kimmel and A. Marciniak-Czochra.

## Background

Cancer research, both experimental and theoretical, in last 12 years has been deeply influenced by the paper “The Hallmarks of Cancer” by [1]. In this paper six key aspects (“hallmarks”) of cancer development and growth were identified and examined. Their interplay with other cellular populations was mainly seen as cooperative, and thus positive for the tumour. Recently, the same authors have published an interesting follow-up paper, “Hallmarks of Cancer: The Next Generation” [2], where their description of cancer development and growth was modified and “up-dated”. In particular, among the various new topics discussed, two important new aspects were added: the role of epigenetic phenomena and the possibility of competitive interplay with the innate and adaptive immune systems. In particular, evasion from immune destruction is explicitly listed as a new “hallmark”.

Tumour cells are characterized by a large number of genetic and epigenetic events leading to the appearance of specific antigens (e.g. mutated proteins, under/over-expressed normal proteins and many others) triggering reactions by the both the innate and the adaptive immune system [3-7]. These observations have provided a theoretical basis to the empirical hypothesis of immune surveillance, i.e. that the immune system may act to eliminate tumours [8], only recently experimentally and epidemiologically confirmed [9]. Of course, the competitive interaction between tumour cells and the immune system involves a considerable number of events and molecules, and as such is extremely complex.

Moreover, to describe fully these immuno-oncological dynamics, one has to take into account a range of spatial phenomena, which are of outmost relevance in determining the dynamics of both immunogenic and non-immunogenic tumours [10-12]. In particular, the interplay between tumour cells and the immune system is strongly influenced by the spatial mobility of both tumour cells and cells of the immune system i.e. effector cells [13]. Apart from the random motion of both types of cell, a prominent role is played by chemoattraction of effector cells towards the tumour cells. Indeed, chemotactic motion of immune system cells is a hallmark of the defence of the human body against “non-self agents”, including tumours, since cells belonging to both the innate immune system (e.g. Natural Killers, Macrophages, Dendritic Cells, etc. [3]) and adaptive immune system (e.g. Cytotoxic T Lymphocites, etc. [3]) are able to reach their targets thanks to the gradients of various kinds of chemicals [3,14], e.g. inflammation-related substances produced by tumour cells. Thus, chemotaxis is of paramount importance in the interplay between tumours and the immune system, since it influences the control of tumour growth and also the immune surveillance.

However, besides temporal and spatial non-linearities, another important point to stress is that the structure of the above-mentioned interactions is also characterized by a series of evolutionary phenomena. As is self-evident, the immune system is not able to eliminate all neoplasms. In other cases, a dynamic equilibrium may also be established, such that the tumour may survive in a so-called “dormant state” [15-18], which is undetectable (i.e. the tumour persists at a very low, undetectable level of cells but is not completely eliminated by the immune system). Until recently this was largely inferred from clinical data, but [15] have been able to show experimentally, through an *ad hoc* mouse model, that adaptive immunity can maintain an occult cancer in an equilibrium state. It is quite intuitive that this equilibrium can be disrupted by sudden events affecting the immune system. If disease-related impairments of the innate and adaptive immune systems, or immuno-suppressive treatments preceding organ transplantations occur, then tumour regrowth occurs [9,19]. This has been shown both by mouse models and through epidemiological studies [9,19].

However, there is a major class of causes of disruption of the equilibrium that is not related to immuno-suppression. Over a long period of time [9], a neoplasm may develop multiple strategies to circumvent the action of the immune system [5,9], which may allow it to recommence growing [9,18] into clinically apparent tumours [15], which theoretically can reach their maximum carrying capacity [18]. From an ecological point of view, we could say that the tumour has adapted to survive in a hostile environment, in which the anti-tumour immune response is activated [9,18]. For example, the tumour may develop mechanisms to grow and spread by reducing its immunogenicity [5,9]. In other words, the immunogenic phenotype of the tumour is influenced by the interaction with the immune system of the host. For this reason, the theory of the interactions between a tumour and the immune system has been called immuno-editing theory [9].

An impressive body of research is accumulating on immuno-evasive strategies, and a recent monograph [20] has been devoted to some aspects of this fascinating subject and to its close relationship with the effectiveness of immunotherapies. As far as the mathematical modelling of tumour and immune system interactions is concerned, there are many papers in the current literature which use deterministic models [13,17,18,21-30] or stochastic models [31-35], as well as models introduced by Bellomo based on the kinetic theories of nonlinear statistical mechanics [36,37]. The general approach of Bellomo’s theory is based on the concept of changes of activities of both the tumour cells and the effector cells of the immune system after encounters between them. As far as spatial aspects are concerned, [38,39] developed a detailed spatio-temporal model focused on the role of macrophages. They showed that the presence of chemoattraction of macrophages towards the tumour cells implies both the onset of traveling waves and a heterogeneous spatial distribution of the tumour cells (see also [40]). Matzavinos, Chaplain and Kuznetsov proposed a spatiotemporal model of the interactions between tumour cells and cytotoxic T-lymphocytes (CTLs) [13,16] by including the spatial motility of both tumour cells and CTLs, as well as chemotactic motion of the CTLs. They focused mainly on the role of the immune system in determining dormant states of the tumour, by showing, through a series of simulations, that a dormant state is reached, but the tumour cells are spatially distributed in an irregular pattern, which also temporally oscillates in a non-periodic fashion (see also [40]). In [18,28], the immuno-editing phenomenon was empirically modelled by allowing the presence of slowly time-varying generic parameters in deterministic models (with time-scales significantly longer than those typical of the tumour-immune system interaction). Recently, in the framework of the above-mentioned kinetic approach, a generic model has been proposed for the learning ability of effector cells and for the hiding of tumour cells [41]. In this paper, based on the concept introduced by [9] that the immune system has the ability of *“sculpting the phenotype”*[9] of tumour cells (i.e. promoting the change towards less imunogenic and more resistant phenotypes), we propose a cell-centered semi-mechanistic approach aimed at describing a possible immunologically realistic kinetic mechanism through which immuno-evasion begins. Since there is strong experimental evidence that type, density and location of CTLs are predictive of the clinical outcome of some tumours, such as colorectal tumours [42], and since we are interested in the long-time dynamics, here we shall deal with the interplay of a neoplasm with CTLs. In our model, we suppose that the tumour cells that survive an attack by CTLs have a probability of acquiring (through mutations or even by epigenetic changes) a phenotype that is more resistant to future attacks by CTLs. In turn, at each new encounter with a CTL, this resistance can be increased further, and after a finite number of encounters a complete or *maximal* resistance to specific immunity is acquired. Moreover, specific spatial effects may be linked to the immuno-evasion of neoplasms. Indeed, recently [43] showed experimentally that tumour cells can produce chemical CXCL12 that, for large concentrations, act as chemorepellent for CTLs, whereas for low concentration it acts as a normal chemoattractor. We integrate - with some simplifications - these experimental findings in our model by permitting in the range of features defining the increasingly resistant tumour cell phenotypes an increasing ability to produce such chemorepulsive substances, whereas in a future model we shall consider the above described “nonmonotone” behavior of the taxis. These two bio-theoretical hypotheses, although new, are in line with the general schema of tumour cell escape from the immune response. Indeed, as stressed by [19], tumour cells may escape from immune control through two general mechanisms: *(a)* mechanisms that involve the secretion of soluble factors; *(b)* mechanisms that are dependent on the contact between the tumour cells and the effectors and that are aimed at reducing antigen recognition/adhesion and apoptotic resistance. Given the current experimental knowledge the above mentioned factors are primarily aimed - apart from, in many cases, their mitogenic action - at inducing the emergence of immunosuppressive networks [44]. In our present model, the factors, in line with the animal model by [43], are chemicals that repel CTLs. Finally, in the concluding remarks, we shall also discuss, from and evolutionary point of view, the differences of our model with the current evolutionary view of the immuno-editing.

## Methods

### The Mathematical Model

**Figure 1:**
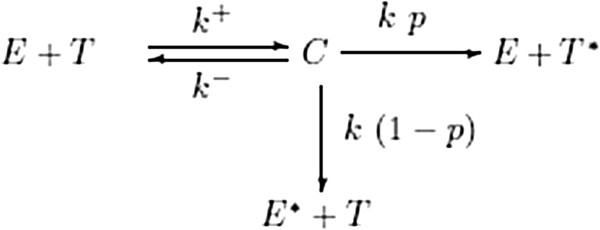
**Basic local lymphocyte-cancer cell interactions.** Schematic diagram of the basic local lymphocyte-cancer cell interactions.

Following the kinetic scheme employed by [13], in absence of immuno-editing mechanisms the interplay between tumour cells and tumour-infiltrating cytotoxic-T-lymphocytes can be modelled as shown in Figure 1 (see: [13]), where *T* denotes a tumour cell, *E* denotes an effector cell (CTL), *C* denotes the complex formed, *T*^∗^denotes a dead tumour cell and *E*^∗^denotes a dead effector cell. The following assumptions are made: 

  the complexes *C* consist of a tumour cell and a CTL forming at a rate *k*^ + ^. The parameter *k*^ + ^consists of the encounter rate between a tumour cell and a CTL, the probability that the CTL recognizes the tumour cell as a “non-self” entity, and also the probability that the tumour cell forms a complex with the CTLs

  the break-up of complexes can lead to a situation where both the tumour cell and the CTLs are alive with a rate *k*^−^

  the break-up of complexes can lead to a situation where either the immune cell or the tumour cell survives the encounter with a rate *k*

  the probability that a tumour cell is killed is *p*, and correspondingly the probability that a CTL is killed (i.e. the tumour cells survives) is (1−*p*)

Using the Law of Mass Action, this leads to the following system of differential equations describing these specific kinetic interactions:

(1) $$\begin{array}{lll} \partial_{t}C & =k^{+}\text{ET}-(k^{-}+k)C\; & \;\\ \partial_{t}T & =-k^{+}\text{ET}+k^{-}C+k(1-p)C\;\\ \partial_{t}E & =-k^{+}\text{ET}+k^{-}C+\text{kpC}\; & \; \end{array}$$

The key idea proposed in this paper which develops the work of [13], is that a proportion of the tumour cells that survive an encounter with a CTL are more resistant to any future attacks by CTLs. Consequently, the phenotypic properties of these new “enhanced” tumour cells will be different from those of the “naive” tumour cells. Specifically, we make the additional assumptions: 

  their probability of being killed (previously the parameter *p*) is smaller

  their probability of being recognized and also of forming a complex with a CTL (embedded in the parameter *k*^ + ^) is smaller

Moreover, we shall also assume that the proliferation rate of CTLs stimulated by the presence of the complexes is also smaller. We denote the naive tumour cells by *T*_0_(*t*) and the non-naive tumour cells by *T*_*i*_, where *i* stands for the number of previous encounters with the CTLs. We assume that the fitness of tumour cells increases up to a maximum number of encounters *N*, implying that we consider in total 1 + *N* “classes” of tumour cells, *T*_0_,*T*_1_,…,*T*_*N*_.

**Figure 2:**
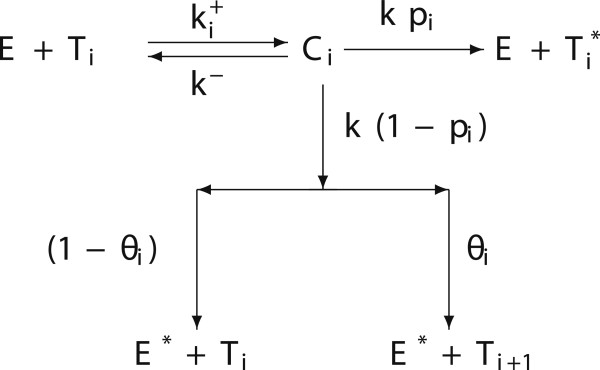
**Extended local lymphocyte-cancer cell interactions.** Schematic diagram of the extended local lymphocyte-cancer cell interactions.

The new kinetic relationships of our model are illustrated in Figure 2 and are characterized by the following new groups of parameters: 

  the rate of formation of complexes [*ET*_*i*_]: $\left(k_{i}^{+}\right)$. We assume that $\left(k_{i}^{+}\right)$ is constant or decreasing with index *i*, with $\left(k_{N}^{+}\geq 0\right)$;

  the probability that a tumour cell of the *i*-th class is killed: *p*_*i*_. We assume that *p*_*i*_is decreasing with index *i*, with *p*_*N*_≥0;

  the probability of transition *T*_*i*_→*T*_*i* + 1_to the state *i*: *θ*_*i*_. We assume that *θ*_*i*_is increasing for 0≤*i*≤*N*−1. Since we have assumed *N* classes of tumour cells, *θ*_*N*_=0.

As far as the temporal dynamics of the tumour cells, CTLs and complexes is concerned, once again using the Law of Mass Action, the kinetic scheme of Figure 2 can be translated into the following system of ordinary differential equations:

(2) $$\begin{array}{lll} \frac{\partial T_{0}}{\mathrm{\partial t}}\;\; & =\;\;-k_{0}^{+}ET_{0}+k^{-}C_{0}+k(1-\theta_{0})(1-p_{0})C_{0}\; & \;\\ \frac{\partial T_{i}}{\mathrm{\partial t}}\;\; & =\;\;-k_{i}^{+}ET_{i}+k\theta_{i-1}(1-p_{i-1})C_{i-1}\; & \;\\ & \;+(k^{-}+k(1-\theta_{i})(1-p_{i}))C_{i}\; & \;\\ \frac{\partial C_{l}}{\mathrm{\partial t}}\;\; & =\;\;k_{l}^{+}ET_{l}-(k^{-}+k)C_{l}\; & \;\\ \frac{\mathrm{\partial E}}{\mathrm{\partial t}}\;\; & =\;\;-E\left(\overset{N}{\underset{j=0}{\sum }}k_{j}^{+}T_{j}\right)+\left(\overset{N}{\underset{j=0}{\sum }}k^{-}C_{j}\right)+\left(\overset{N}{\underset{j=0}{\sum }}kp_{j}C_{j}\right)\; & \;\\ & \; \end{array}$$

where *i*=1,…,*N*, and *l*=0,…,*N*.

However, not only the temporal but also the spatio-temporal properties of the “fitter” tumour cells are likely to be different from those of the baseline tumour cells. Namely: 

  the production rates of chemoattractants stimulated by a complex CTL+’non naive tumour cell’ is assumed to be smaller than that of the naive cells

  since recently Vianello et al. [43] showed in an animal model that tumours produce chemicals that repels the CTLs, here we assume that those chemorepellents are produced by the non-naive cells. For the sake of the precision, Vianello’s findings showed chemoattraction for low concentrations of chemical CXCL12 emitted by tumour cells. For the sake of simplicity here we shall only consider chemorepulsion.

In the following, we provide the full equations for all variables, including the spatial components. As mentioned in the previous section, we have assumed the model of [13] as our baseline model.

#### Spatiotemporal Dynamics of the Tumour Cells

Following [13], we assume that the tumour growth may be described by a logistic law, and that the tumour cells migrate randomly. Thus, it follows that the spatio-temporal dynamics of the naive tumour cells *T*_0_ is as follows:

(3) $$\begin{array}{lll} \frac{\partial T_{0}}{\mathrm{\partial t}}\;\;= & \;\;\overset{\text{random}\;\text{motion}}{\overset{︷}{D_{T_{0}}\nabla^{2}T_{0}}}+\overset{\text{logistic}\;\text{growth}}{\overset{︷}{r_{1}T_{0}\left(1-\beta_{1}\overset{N}{\underset{j=0}{\sum }}T_{j}\right)}}\; & \;\\ & -\overset{\text{local}\;\text{kineticsl}}{\overset{︷}{k_{0}^{+}ET_{0}+(k^{-}+k(1-\theta_{0})(1-p_{0}))C_{0}}}\; & \; \end{array}$$

and the dynamics of the non-naive cells *T*_*i*_is given by:

(4) $$\begin{array}{lll} \frac{\partial T_{i}}{\mathrm{\partial t}}\;\; & =\;\;\overset{\text{random}\;\text{motion}}{\overset{︷}{D_{T_{i}}\nabla^{2}T_{i}}}+\overset{\text{logistic}\;\text{growth}}{\overset{︷}{r_{1}T_{i}\left(1-\beta_{1}\overset{N}{\underset{j=0}{\sum }}T_{j}\right)}}\; & \;\\ & \;\;\;-\overset{\text{local}\;\text{kineticsl}}{\overset{︷}{k_{i}^{+}ET_{i}+(k^{-}+k(1-\theta_{i})(1-p_{i}))C_{i}+k\theta_{i-1}(1-p_{i-1})C_{i-1}}}\; & \; \end{array}$$

where *i*=1,…,*N*, and where *r*_1_ is the baseline exponential growth rate of the tumour (i.e. its theoretical growth rate when it is ‘small’) and *β*_1_ is the inverse of its carrying capacity (in absence of immune reactions).

#### Spatiotemporal Dynamics of the CTLs

Considering the CTLs, as in [13], both random and chemotactic motion of these cells is included. However, as previously discussed, an additional type of motility is included due to the postulated onset of “negative taxis” due to the production of a chemorepellent *ρ*by the non-naive tumour cells. This results in the following equation:

(5) $$\begin{array}{lll} \;\frac{\mathrm{\partial E}}{\mathrm{\partial t}}\;\;= & \;\;\overset{\text{random}\;\text{motility}}{\overset{︷}{D_{E}\nabla^{2}E}}-\overset{\text{chemotaxis}}{\overset{︷}{\chi (\alpha )\nabla .(E\nabla \alpha )}}\; & \;\\ & \;+\;\overset{\text{chemorepulsion}}{\overset{︷}{A(\rho )\nabla .(E\nabla \rho )}}+\overset{\text{supply}}{\overset{︷}{\text{sh}(x)}}+\overset{\text{proliferation}}{\overset{︷}{\frac{f\overset{N}{\underset{j=0}{\sum }}q_{j}C_{j}}{g+\overset{N}{\underset{j=0}{\sum }}T_{j}}}}\; & \;\\ & \;-\;\overset{\text{decay}}{\overset{︷}{d_{1}E}}\;-\;\overset{\text{local}\;\text{kineticsl}}{\overset{︷}{E\left(\overset{N}{\underset{j=0}{\sum }}k_{j}^{+}T_{j}\right)\;\;+\;\;\left(\overset{N}{\underset{j=0}{\sum }}k^{-}C_{j}\right)\;\;+\;\;\left(\overset{N}{\underset{j=0}{\sum }}kp_{j}C_{j}\right)}}\; & \; \end{array}$$

The proliferation of the CTLs, *E*, stimulated by the complexes *C*_*j*_ is embedded in the rate constant *q*_*j*_, which, as a consequence, must be decreasing with the index *j*, with *q*_*N*_≥0 (*f *, *g* are constant parameters). Note that in absence of immuno-editing this proliferation term reads *fC*/(*g* + *T*), and it has been has been introduced in [22,45]. It represents the experimentally observed enhanced proliferation of CTLs in response to the tumour. This functional form is consistent with a model in which one assumes that the enhanced proliferation of CTLs is due to signals, such as released interleukins, generated by effector cells in tumour cell-CTL complexes. We note that the growth factors that are secreted by lymphocytes in complexes (e.g IL-2) act mainly in an autocrine fashion. That is to say they act on the cell from which they have been secreted and thus, in our spatial setting, their action can be adequately described by a “local” kinetic term only, without the need to incorporate any additional information concerning diffusivity. The external influx of CTLs is, for the sake of the simplicity, modelled as *sh*(*x*), where *h*(*x*) is a Heaviside function, taken to be zero over a given subregion of the domain of interest cf. [13]. In other words, we assume that there is a subdomain where lymphocytes are not naturally present and which is penetrated by CTLs only thanks to diffusion and chemotaxis.

#### Chemoattractant

The spatiotemporal dynamics of the chemoattractant *α*produced by the complexes is given by

(6) $$\begin{array}{lll} \frac{\partial \alpha }{\mathrm{\partial t}}\;\; & =\;\;\overset{\text{diffusion}}{\overset{︷}{D_{2}\nabla^{2}\alpha }}-\overset{\text{decay}}{\overset{︷}{\delta_{1}\alpha }}+\overset{\text{production}}{\overset{︷}{\left(\overset{N}{\underset{j=0}{\sum }}\Pi_{j}C_{j}\right)}}\; & \; \end{array}$$

where we assume that the production rate constant *Π*_*i*_is decreasing with index *i*, with *Π*_*N*_≥0, since we assume that complexes between CTLs and non-naive tumour cells are less and less able to produce such a chemoattractant.

#### Chemorepellent

Adapting the experimental findings by Vianello to our framework, we suppose that the non-naive tumour cells produce a chemical that repels the CTLs and whose concentration *ρ* is governed by the equation

(7) $$\begin{array}{lll} \frac{\partial \rho }{\mathrm{\partial t}}\;\; & =\;\;\overset{\text{diffusion}}{\overset{︷}{D_{*}\nabla^{2}\rho }}-\overset{\text{decay}}{\overset{︷}{\delta_{2}\rho }}+\overset{\text{production}}{\overset{︷}{\left(\overset{N}{\underset{j=0}{\sum }}w_{j}T_{j}\right)}}\; & \; \end{array}$$

where the production rate constants *w*_*i*_are such that: *w*_0_=0 (absence of production for naive tumour cells) and

(8) $$w_{1}<w_{2}<\cdots <w_{N}.$$

#### Tumour cell-CTL Complexes

Following [13], we assume that the motility of the complexes is so small that it can be neglected:

(9) $$\begin{array}{lll} \frac{\partial C_{l}}{\mathrm{\partial t}}\;\; & =\;\;\overset{\text{local kinetic}}{\overset{︷}{k_{l}^{+}ET_{l}-(k^{-}+k)C_{l}}}\; & \; \end{array}$$

where *l*=0,…,*N*.

#### Modeling the transition rates and probabilities

Concerning the transitions *T*_*i*_→*T*_*i* + 1_, we assume that they are a linear function of *i*:

(10) $$\begin{array}{lll} & \theta_{i}=\theta_{0}+(\theta_{\text{MAX}}-\theta_{0})\frac{i}{N-1},\;i=0,\ldots ,N-1\; & \;\\ & \;\;\;\theta_{N}=0,\;\theta_{\text{MAX}}=10\theta_{0}.\; & \; \end{array}$$

and that their baseline value is sufficiently small: 10^−5^≤*θ*_0_≤10^−3^. In other words we assume that the probability of acquiring the less immunogenic phenotype is small (in analogy with the smallness of the probability of surviving to an attack by a CTLs).

The probability *p*_*i*_ that a tumour cell of class *T*_*i*_is lethally hit is given by:

(11) $$p_{i}=p_{0}+(p_{N}-p_{0})\frac{i}{N},$$

where 0≤*p*_*N*_<*p*_0_. Concerning the rates $\left(k_{i}^{+}\right)$, we assume either that they are constant or that they are linearly decreasing with $\left(k_{N}^{+}=0\right)$:

(12) $$k_{i}^{+}=k_{0}^{+}\left(1-\frac{i}{N}\right).$$

The production rate of the chemoattractant is also assumed to vary linearly:

(13) $$\Pi_{i}=\Pi_{0}\left(1-\frac{i}{N}\right)$$

with [16]: *Π*_0_=20−3000*moleculescells*^−1^*min*^−1^, We suppose that the chemorepellent is produced via a mechanism of “threshold generation”, i.e. only after a sufficient number of encounters, yielding:

(14) $$w_{i}=\left\{\begin{array}{c} 0,\;\;\;\;\text{if}\;0\leq i\leq N_{*},\\ w_{\text{MAX}}\frac{i-N_{*}}{N-N_{*}},\;\;\;N_{*}<i\leq N \end{array}\right)$$

where we assumed: *w*_*MAX*_≈*Π*_0_(i.e. the maximum production rate of the chemorepellent is equal to the production rate of the chemoattractant in absence of immuno-editing phenomena).

### Boundary and initial conditions

We initially consider the model in a fixed 1-dimensional domain [0,*x*_*a*_] and close the system by applying appropriate boundary and initial conditions. As far as the boundary conditions are concerned, zero-flux boundary conditions are imposed on all state variables (apart from *C*): *E*, *α*, *ρ* and *T*_*i*_, *i*=0,…,*N*. These boundary conditions are appropriate for the tumour-immune dynamics we are considering. For example, in *BCL*_1_lymphomas of the spleen tumour cells are spatially contained in the lymph tissue of the spleen, an elongated organ that, in mice, is characterized by a very strong basement membrane, which is only broken when the tumour cells switch to an “invasive phenotype”. Since here we are concerned with earlier stage dynamics of tumour cells in a dormant state evading the CTLs, it follows that the zero-flux boundary conditions are adequate for our particular model. As far as the initial conditions are concerned, we assume an initial front of naive tumour cells encountering a front of CTLs, resulting in the formation of *C*_0_complexes. We suppose that initially there are no non-naive tumour cells and hence no complexes involving them. No chemicals are initially present in the spatial domain. These assumptions yield:

(15) $$\;\begin{array}{ll} E(x,0) & =\left\{\begin{array}{ll} 0, & \;\text{if}\;0\leq x\leq l,\\ E_{a}(1-\text{exp}(-1000{\left(x-l\right)}^{2})), & \;\text{if}\;l<x\leq x_{a}. \end{array}\right)\\ T_{0}(x,0) & =\left\{\begin{array}{ll} T_{a}(1-\text{exp}(-1000{\left(x-l\right)}^{2})), & \;\text{if}\;0\leq x\leq l,\\ 0 & \;\text{if}\;l<x\leq x_{a}. \end{array}\right)\\ C_{0}(x,0) & =\left\{\begin{array}{ll} 0, & \;\text{if}\;x\notin [l-\varepsilon ,l+\varepsilon ],\\ C_{a}\text{exp}(-1000{\left(x-l\right)}^{2}), & \;\text{if}\;x\in [l-\varepsilon ,l+\varepsilon ]. \end{array}\right)\\ & \;T_{i}(x,0)=0,\;C_{i}(x,0)=0,\forall x\in [0,x_{a}].\\ & \;\;\;\;\alpha (x,0)=0,\;\rho (x,0)=0,\;\forall x\in [0,x_{a}]. \end{array}$$

where

(16) $$\begin{array}{lll} & E_{a}=\frac{s}{d_{1}},\;\;T_{a}=\frac{1}{\beta_{1}},\;\;C_{a}=\min(E_{a},T_{a}),\;\;0<\varepsilon \ll 1,\; & \;\\ & i=1,\ldots ,\mathrm{N.}\; & \; \end{array}$$

Note that *E*_*a*_is the baseline homogenous steady-state value of CTLs in the absence of tumour cells, and that *T*_*a*_is the the baseline homogenous steady-state value of the naive cells in absence of CTLs, whereas *C*_*a*_ is the maximum possible density of complexes resulting from initial values of *E* and *T*.

## Results and Discussion

We simulated our system after nondimensionalizing it as shown in the Appendix, where one can also find the numerical values of the parameters. In addition to the baseline parameter set detailed in the Appendix, in the following sections all our simulations were performed assuming the following values for key parameters associated with the encounter of CTLs and tumour cells:

(17) $$\theta_{0}=10^{-4},\;p_{N}\in \{0,0.5,0.75,0.9997\}.$$

Moreover $\left(k_{0}^{+}=1.3\times 10^{-7}\right)$[13] and $\left(k_{i}^{+}\right)$ may be either constant or linearly decreasing, with $\left(k_{N}^{+}=0\right)$.

Since the average lifespan of a chimeric mouse is three years, and since we are interested in assessing the possibility (and spatio-temporal modality) of the onset of immunoevasion of a tumour, all simulations (unless stated) represent an interval of length 1100 days ≈3 years.

### Spatially Homogenous Case

**Figure 3:**
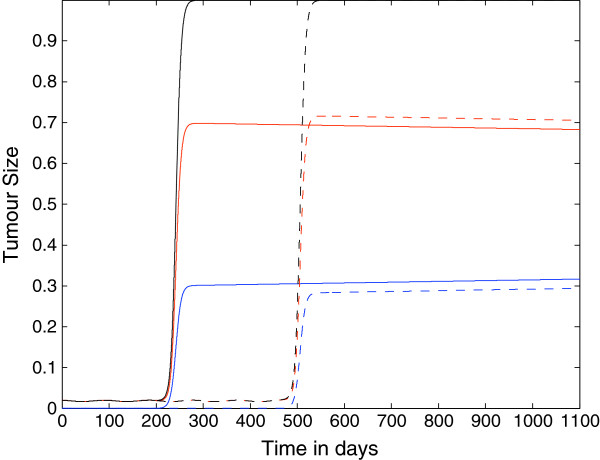
**Growth of the tumour: nonspatial case 1.** Plots showing the growth of the tumour cell population over time in the case where the spatial components of the model (i.e. all diffusion, taxis terms) have been set to zero. The plots show that the tumour can evade the immune system for either approximately 200 days or approximately 500 days depending on the parameter *N*. Parameter values: *p*_*N*_=0 and $\left(k_{i}^{+}=\text{constant}=1.3\times 10^{-7}\right)$ and: *N*=4 (solid line) and *N*=10 (dashed lines). The red lines represent the population *T*_0_, the blue lines represent the summed populations *T*_1_ + … + *T*_*N*_, and the black lines represent the summed populations *T*_0_ + … + *T*_*N*_. Time *t* is in days.

In this first set of simulations, we set all the spatial components of the model to zero and consider only the reaction kinetics in order to ascertain whether the primary mechanism of evasion can be purely temporal. All simulations suggest that our model, with the parameter assumptions and values we used, is able to reproduce the onset of immunoevasion in a biologically realistic time-frame. Figure 3 shows the plots of the growth of the tumour cell population over time where the killing probability at the last stage is zero, i.e. *p*_*N*_=0, and $\left(k_{i}^{+}=\text{constant}=1.3\times 10^{-7}\right)$. We observe that if *N*=4 the onset of evasion is at *t*≈200 days, i.e. the tumour remains dormant for 200 days, which is a long period of time for a mouse. On the contrary, if *N*=10 then the immunoevasion is delayed even further, with onset at *t*≈500 days.

**Figure 4:**
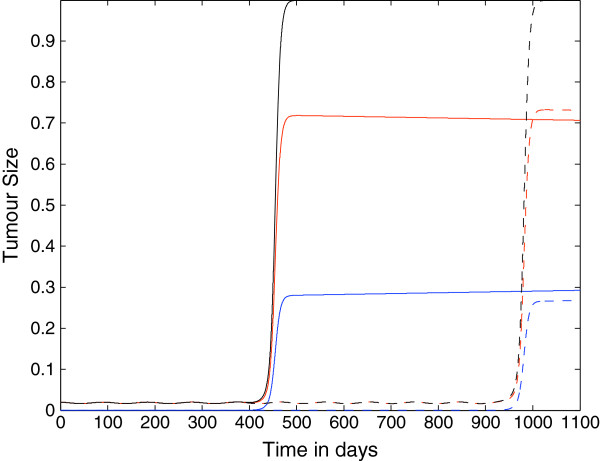
**Growth of the tumour cell population: nonspatial case 2.** Plots showing the growth of the tumour cell population over time in the case where the spatial components of the model (i.e. all diffusion, taxis terms) have been set to zero. The plots show that the tumour can evade the immune system for either approximately 400 days or approximately 950 days depending on the parameter *N*. Parameter values: *p*_*N*_=0.5 and $\left(k_{i}^{+}=\text{constant}=1.3\times 10^{-7}\right)$ and: *N*=4 (solid line) and *N*=10 (dashed lines). The red lines represent the population *T*_0_, the blue lines represent the summed populations *T*_1_ + … + *T*_*N*_, and the black lines represent the summed populations *T*_0_ + … + *T*_*N*_. Time *t* is in days.

Figure 4 shows the plots of the growth of the tumour cell population over time where the killing probability at the last stage is not zero but it is only halved, i.e. *p*_*N*_=0.5, and as in the previous figure, $\left(k_{i}^{+}=\text{constant}=1.3\times 10^{-7}\right)$. Also in this case the immunoevasion is reproduced and takes place, respectively, at *t*≈425 *days* for *N*=4 and at *t*≈950 *days* for *N*=10.

**Figure 5:**
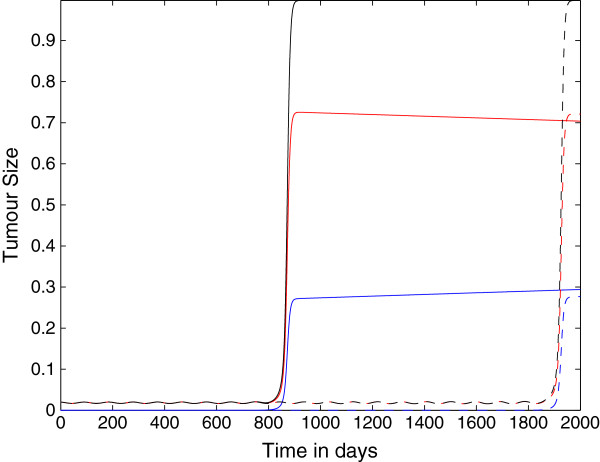
**Growth of the tumour cell population: nonspatial case 3.** Plots showing the growth of the tumour cell population over time in the case where the spatial components of the model (i.e. all diffusion, taxis terms) have been set to zero. The plots show that the tumour can evade the immune system for either approximately 850 days or approximately 1900 days depending on the parameter *N*. Parameter values: *p*_*N*_=0.75 and $\left(k_{i}^{+}=\text{constant}=1.3\times 10^{-7}\right)$ and: *N*=4 (solid line) and *N*=10 (dashed lines). The red lines represent the population *T*_0_, the blue lines represent the summed populations *T*_1_ + … + *T*_*N*_, and the black lines represent the summed populations *T*_0_ + … + *T*_*N*_. Time *t* is in days.

Figure 5 shows the plots of the growth of the tumour cell population over time where the killing probability at the last stage is *p*_*N*_=0.75, and as in the previous figure, $\left(k_{i}^{+}=\text{constant}=1.3\times 10^{-7}\right)$. These results are different from the previous two cases. Here the immunoevasion takes place *in the lifespan of the mouse* only for the case *N*=4. This suggests that in absence of changes in the parameter $\left(k_{i}^{+}\right)$: *i)* the late stages *T*_*i*_are the most important to determine the onset of the evasion; *ii)* due to the finite lifespan of chimeric mice and to the slow rate of the transitions, the immunoevasion process requires that the maximum ability of genetic or epigenetic changes in a tumour cell upon forming a complex with a CTL (embedded in the transition probability whose maximum, we recall, is at *i*=*N*−1), is reached in a small number of encounters.

**Figure 6:**
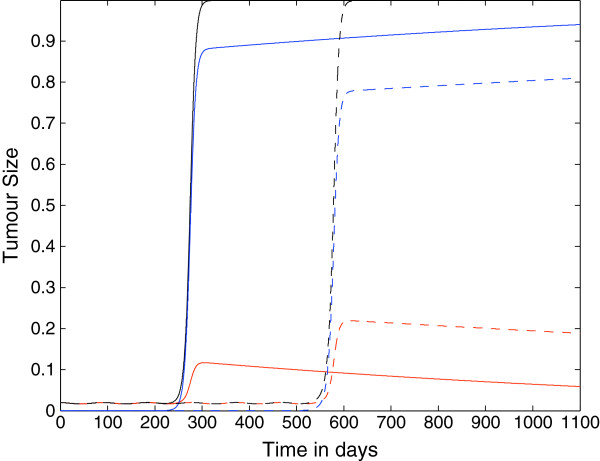
**Growth of the tumour cell population: nonspatial case 4.** Plots showing the growth of the tumour cell population over time in the case where the spatial components of the model (i.e. all diffusion, taxis terms) have been set to zero. The plots show that the tumour can evade the immune system for either approximately 250 days or approximately 550 days depending on the parameter *N*. Parameter values: *p*_*N*_=0.75 and $\left(k_{i}^{+}\right)$ are linearly decreasing functions and: *N*=4 (solid line) and *N*=10 (dashed lines). The red lines represent the population *T*_0_, the blue lines represent the summed populations *T*_1_ + … + *T*_*N*_, and the black lines represent the summed populations *T*_0_ + … + *T*_*N*_. Time *t* is in days.

**Figure 7:**
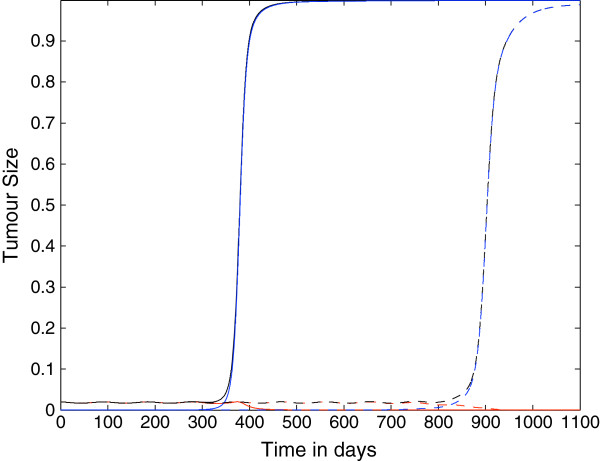
**Growth of the tumour cell population: nonspatial case 5.** Plots showing the growth of the tumour cell population over time in the case where the spatial components of the model (i.e. all diffusion, taxis terms) have been set to zero. The plots show that the tumour can evade the immune system for either approximately 350 days or approximately 850 days depending on the parameter *N*. In this case however, the initial population *T*_0_is eradicated. Parameter values: *p*_*i*_=*constant*=0.9997 and $\left(k_{i}^{+}\right)$ are linearly decreasing functions and: *N*=4 (solid line) and *N*=10 (dashed lines). The red lines represent the population *T*_0_, the blue lines represent the summed populations *T*_1_ + … + *T*_*N*_, and the black lines represent the summed populations *T*_0_ + … + *T*_*N*_. Time *t* is in days.

Figure 6 shows the growth of the tumour cell population over time where the parameter *p*_*N*_=0.75, but in this case the parameters $\left(k_{i}^{+}\right)$ are linearly decreasing with $\left(k_{N}^{+}=0\right)$. We notice the following differences from the previous case: *i)* here the onset of immunoevasion is for *N*=4 at *t*≈250 *days*, i.e. it is considerably accelerated; *ii)* there is the onset of immunoevasion (at *t*≈550 *days*) also for *N*=10. Thus, this simulation suggests that the role of the decrease of the probability that a tumour cell is recognized by a CTL is important for the timing of the onset of immunoevasion. Moreover, the decrease of the parameters $\left(k_{i}^{+}\right)$ alone is sufficient to induce immunoevasion, as suggested in the simulations shown in Figure 7, where *p*_*i*_=*constant*=0.9997 and $\left(k_{i}^{+}\right)$ are linearly decreasing.

**Figure 8:**
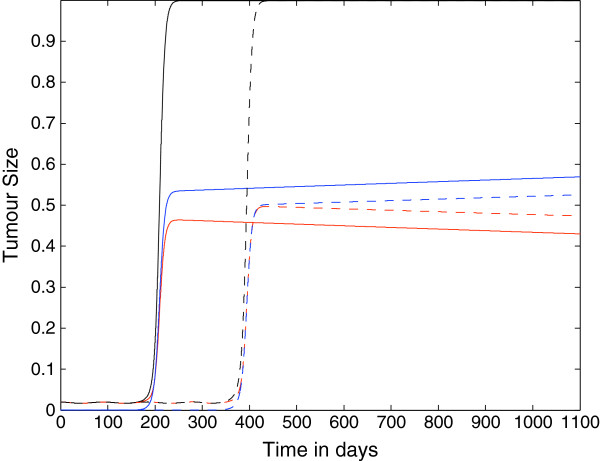
**Nonspatial case 6.** Plots showing the growth of the tumour cell population over time in the case where the spatial components of the model (i.e. all diffusion, taxis terms) have been set to zero. The plots show that the tumour can evade the immune system for either approximately 200 days or approximately 400 days depending on the parameter *N*. Parameter values: *p*_*N*_=0 and $\left(k_{i}^{+}\right)$ are linearly decreasing functions and: *N*=4 (solid line) and *N*=10 (dashed lines). The red lines represent the population *T*_0_, the blue lines represent the summed populations *T*_1_ + … + *T*_*N*_, and the black lines represent the summed populations *T*_0_ + … + *T*_*N*_. Time *t* is in days.

However, as shown in Figure 8, if *p*_*N*_=0, then the addition of the mechanism of decreasing $\left(k_{i}^{+}\right)$ does not accelerate the onset of immunoevasion to such a degree with respect to the baseline case of constant $\left(k_{i}^{+}\right)$ shown in the previous Figure 3.

Finally, comparing the results of our simulations, in the cases where $\left(k_{i}^{+}\right)$ is decreasing we note that the maximum size of the naive tumour cells compartment is smaller than the maximum size of all the non-naive tumour cells compartments summed up:

(18) $$\text{MAX}(T_{0})<\text{MAX}(T_{1}+\cdots +T_{N}).$$

Moreover, *Max*(*T*_0_) seems to be a decreasing function of *p*_*N*_. These results might be explained as follows: the decrease of the competition between all the tumour cells and the immune system embedded in the decrease of the parameters $\left(k_{i}^{+}\right)$, might shift the ‘internal’ competition between the naive and the non-naive tumour cells.

### Spatiotemporal Model

**Figure 9:**
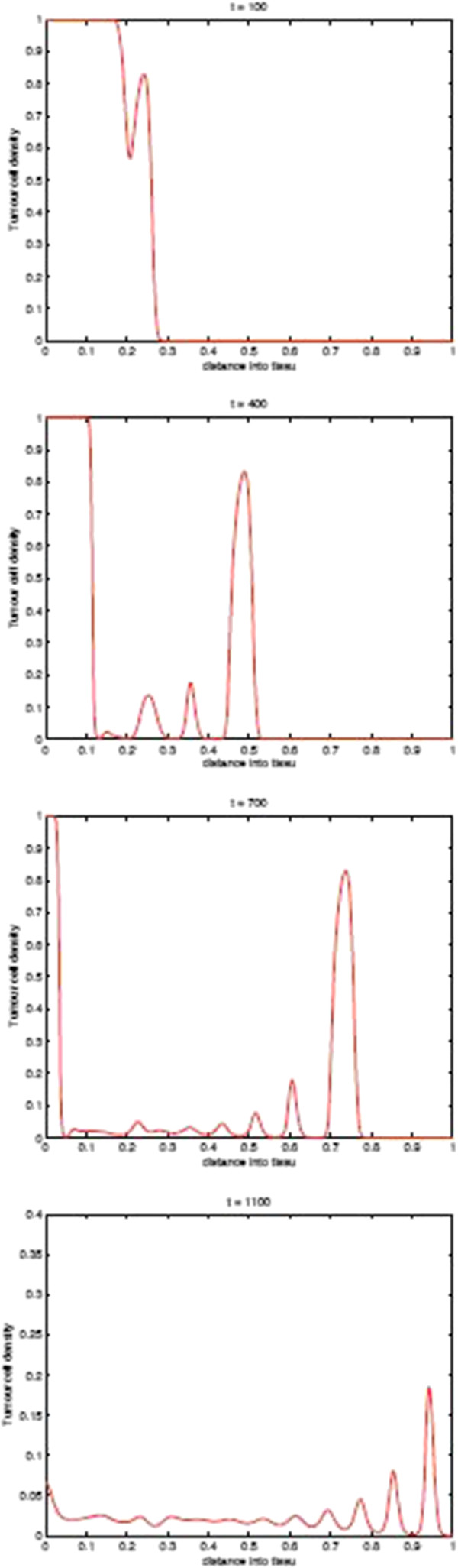
**Tumour Spatial density in absence of immunoevasive mechanisms.** Plots showing the spatial distribution of tumour cells within the tissue at times corresponding to 100, 400, 700, and 1100 days, respectively, in the baseline case of absence of the immunoevasive mechanism described in this paper. This corresponds to the results of [13].

**Figure 10:**
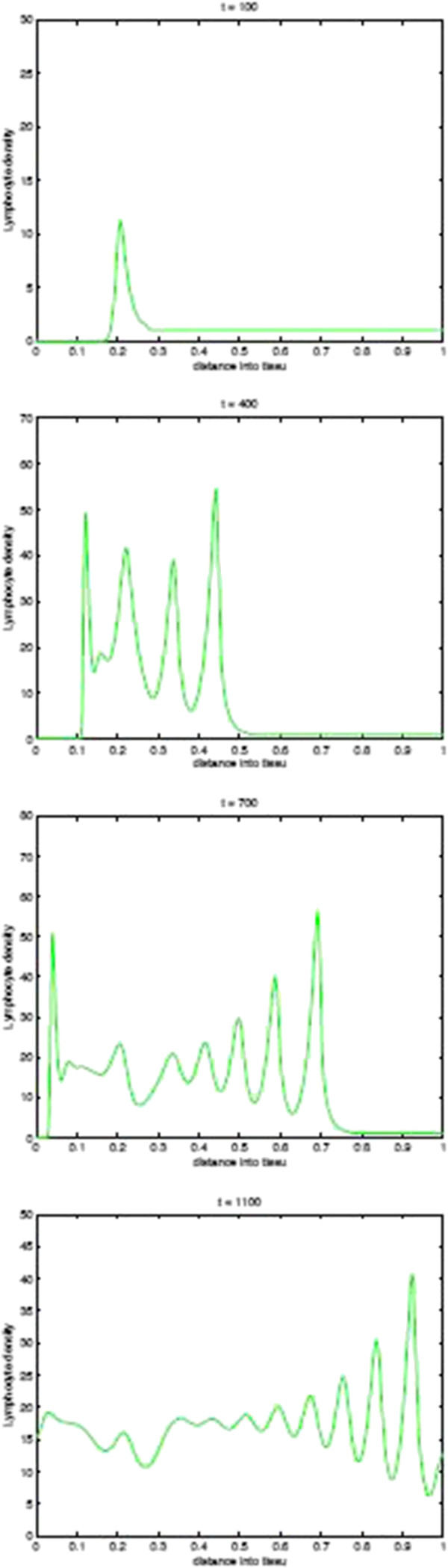
**CTLs Spatial density in absence of immunoevasive mechanisms.** Plots showing the spatial distribution of CTLs within the tissue at times corresponding to 100, 400, 700, and 1100 days, respectively, in the baseline case of absence of the immunoevasive mechanism described in this paper. This corresponds to the results of [13].

Before we present the computational simulation results of the new model in this paper, in Figures 9 and 10 we plot the spatial distribution of tumour cells and CTLs, respectively, in the baseline case of the absence of immunoevasive mechanisms. Figure 9 shows the spatial distribution of tumour cell density within the tissue at times 100, 400, 700, and 1100 days. These results illustrate the basic spatiotemporal dynamics of the tumour cell density induced by its interplay with the distribution of CTLs i.e. a gradual transition between a front of tumour cells to a train of solitary-like travelling waves slowly invading the tissue, finally creating a spatially heterogeneous and time-changing (through irregular temporal oscillations) distribution. Similarly, Figure 10 shows the corresponding plots of the CTL density.

**Figure 11:**
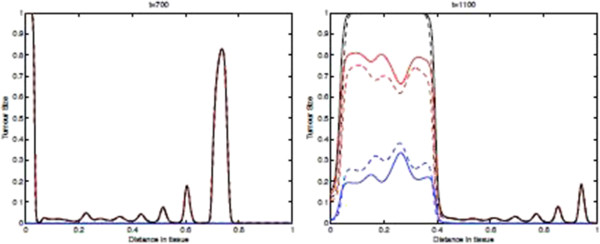
**Tumour Spatial density in presence of immunoevasive mechanisms.** Plots showing the distribution of tumour cell density within the tissue at times corresponding to 700, and 1100 days respectively. These plots illustrate the spatiotemporal onset of immunoevasion. The final plot at *t*=1100 should be compared to the equivalent plot in Figure 9, whereas the plot in the left panel, referring to time *t*=700 *days*, is analogous to the equivalent plot in Figure 9. These plots suggests that the onset of immunoevasion occurs after *t*=700*days*. Parameter values *p*_*N*_=0.75 and $\left(k_{i}^{+}=\text{constant}\right)$. Solid line with chemorepellent, dashed line without. The red lines represent the population *T*_0_, The blue lines represent the summed population *T*_1_ + … + *T*_*N*_, and the black lines represent the summed population *T*_0_ + … + *T*_*N*_.

**Figure 12:**
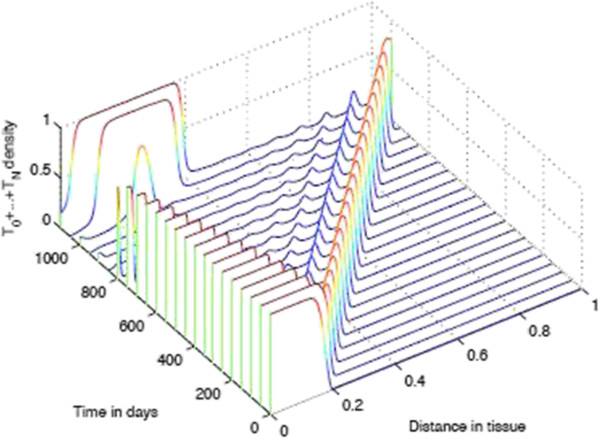
**Time-slices of Tumour Spatial density in presence of immunoevasive mechanisms.** Plots showing detailed changes in the spatial distribution of all tumour cells $\overset{N}{\underset{j=0}{\sum }}T_{j}$ within the tissue over time in the case of immunoevasion. Parameter values *p*_*N*_=0.75 and $\left(k_{i}^{+}=\text{constant}\right)$. This figure shows the onset of immunoevasion in the interval (700,1100) *days*.

Figure 11 shows the spatial distribution of tumour cell density within the tissue at times 700, and 1100 days where the parameters *p*_*N*_=0.75 and $\left(k_{i}^{+}=\text{constant}\right)$. These results show that if we include the immunoevasive mechanism with a decreased value for the parameter *p*_*N*_, we obtain a process that is identical to the baseline case for most of the time. Indeed, basically **the left plot of this figure is identical to that of Figure**9**.** However, after the onset of the evasion, the tumour cell density distribution changes significantly as can be seen in the left part of the domain. From these observed differences, we may say that in this modelling framework the effect of immunoevasion on the spatio-temporal dynamics is characterized by a return to a spatially homogeneous steady-state. This transition to the new spatial regimen is illustrated in more detail by the “time-slices” shown in Figure 12.

Finally, we note that the effect of chemorepulsion, which is well detectable at level of the spatial densities *T*_*i*_, is no more detectable at level of the total density of all tumour cells. However, this effect is again noticeable if we consider the density of *T*_0_ versus the density of *T*_1_ + ⋯ + *T*_*N*_(see the second plot of Figure 11).

**Figure 13:**
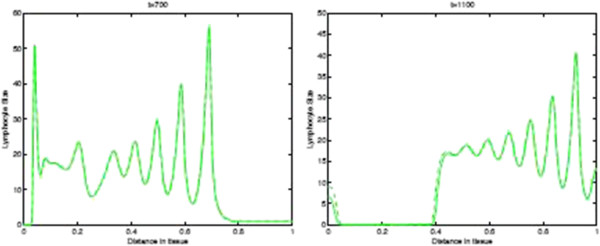
**CTLs Spatial density in presence of immunoevasive mechanisms.** Plots showing the distribution of CTLs within the tissue at times corresponding to 100, 400, 700, and 1100 days respectively. These plots illustrate the spatiotemporal onset of immunoevasion. The final plot at *t*=1100 should be compared to the equivalent plot in Figure 10, whereas the plot in the left panel, referring to time *t*=700 *days*, is analogous to the equivalent plot in Figure 10. These plots suggests that the onset of immunoevasion occurs after *t*=700 *days*. Parameter values *p*_*N*_=0.75 and $\left(k_{i}^{+}=\text{constant}\right)$. Solid line with chemorepellent, dashed line without.

**Figure 14:**
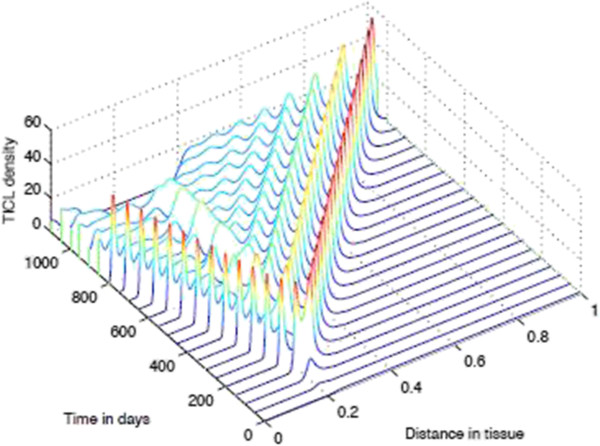
**Time-slices of CTLs in presnece of immunoevasion.** Plots showing detailed changes in the spatial distribution of CTLs within the tissue over time in the case of immunoevasion. Parmeter values *p*_*N*_=0.75 and $\left(k_{i}^{+}=\text{constant}\right)$.

Figures 13 and 14 show the corresponding density of CTLs in the tissue. Note that after the onset of immunoevasion, corresponding to the newly reformed invasive front of tumour cells at a high density, the density of CTLs is close to zero.

**Figure 15:**
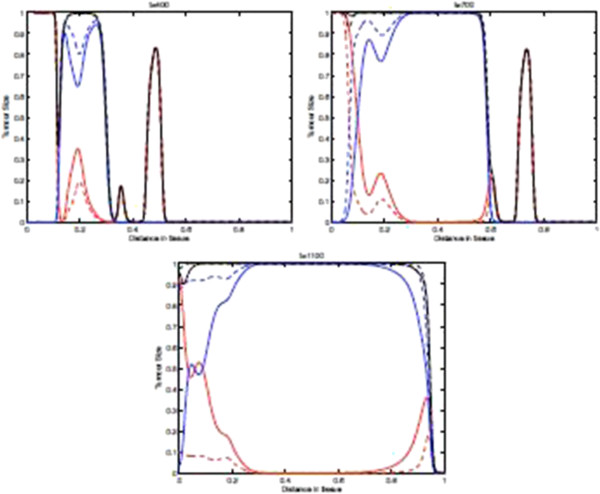
**Tumour cell density within the tissue in for decreasing**$\left(k_{i}^{+}\right)$ and ***p***_***i***_**.** Plots showing the distribution of tumour cell density within the tissue at times corresponding to 400, 700, and 1100 days respectively. These plots illustrate the spatiotemporal onset of immunoevasion. Parameter values *p*_*N*_=0.75 and $\left(k_{i}^{+}\right)$ are decreasing such that $\left(k_{N}^{+}=0\right)$. Solid line with chemorepellent, dashed line without. The red lines represent the population *T*_0_, The blue lines represent the summed population *T*_1_ + … + *T*_*N*_, and the black lines represent the summed population *T*_0_ + … + *T*_*N*_.

Figure 15 shows the spatial distribution of tumour cell density within the tissue at times 400, 700, and 1100 days in the case where the parameters *p*_*N*_=0.75 and $\left(k_{i}^{+}\right)$ are decreasing such that $\left(k_{N}^{+}=0\right)$. Due to the acceleration of the immunoevasion caused by the synergy existing between the variability of *p*_*i*_ and $\left(k_{i}^{+}\right)$, the plots in this figure are substantially different from those in the baseline case in Figure 9 and also with respect to the plots in Figure 11. Indeed, in this case the spatio-temporal distribution of the tumour cell density is far more regular, and by *t*=1100 days almost all of the tissue has been invaded by the tumour cells close to their maximum density. Moreover, here in large regions of the domain we have *T*_0_<*T*_1_ + ⋯ + *T*_*N*_, which is the opposite of the previous case, where the naive tumour cells *T*_0_were prevalent. Finally, Figure 15 illustrates the fact that the distributions of naive vs non-naive tumour cells are “mirror-images” of one another and they are complementary, since their sum is a homogeneous front.

**Figure 16:**
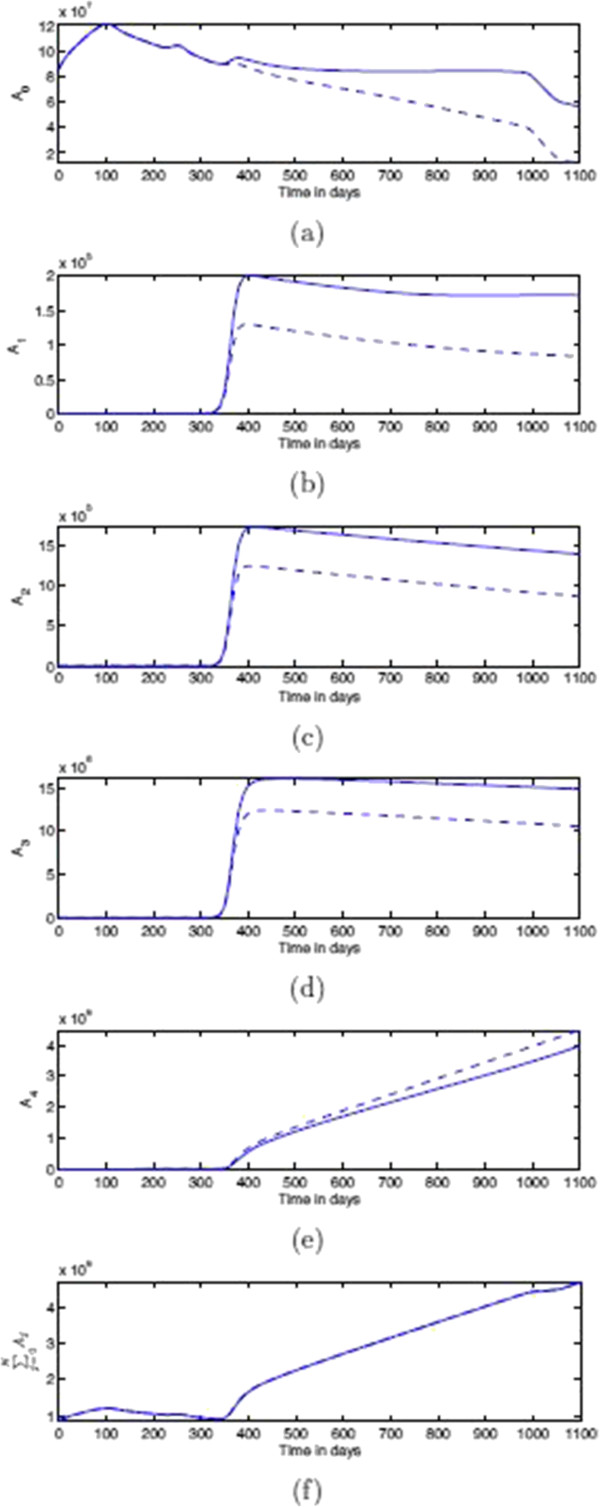
**Effects of chemorepulsion on the total number of spatially distributed classes of tumour cells.** Plots showing the effects of chemorepulsion on the total number of spatially distributed classes of tumour cells. Plots of the total number of cells $A_{i}(t)=\overset{1}{\underset{0}{\int }}T_{i}(x,t)\mathrm{dx}$. Panels: **(a)***A*_0_, **(b)***A*_1_, **(c)***A*_2_, **(d)***A*_3_, **(e)***A*_4_, and **(f)**$\overset{4}{\underset{j=0}{\sum }}A_{j}(t)$. Solid line with chemorepellent, dashed line without. Time is measured in days.

In Figure 16 we show the differential effect of chemorepulsion on the various classes of tumour cells. The plots show the total number *A*_*i*_(*t*) of cells in each classes, i.e.

(19) $$A_{i}(t)=\overset{1}{\underset{0}{\int }}T_{i}(x,t)\mathrm{dx},$$

over time, as well as, in the last plot, the grand-total *A*_1_(*t*) + ⋯ + *A*_*N*_(*t*). The effect of the chemorepulsion on each sub-population *A*_*i*_is striking, although overall it is globally compensated (see the last plot).

**Figure 17:**
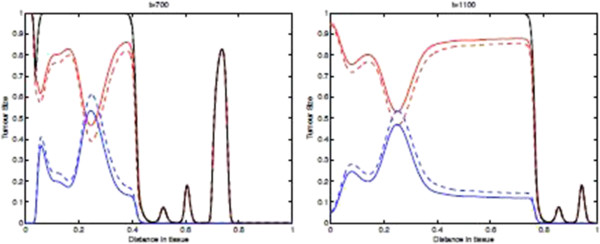
**Distribution of tumour cell density within the tissue for decreasing*p***_***i ***_**and costant**$\left(k_{i}^{+}\right)$**.** Plots showing the distribution of tumour cell density within the tissue at times corresponding to 700, and 1100 days respectively. These plots illustrate the spatiotemporal onset of immunoevasion. Parameter values *p*_*N*_=0.5 and $\left(k_{i}^{+}\right)$ are constant. Solid line with chemorepellent, dashed line without. The red lines represent the population *T*_0_, The blue lines represent the summed population *T*_1_ + … + *T*_*N*_, and the black lines represent the summed population *T*_0_ + … + *T*_*N*_.

**Figure 18:**
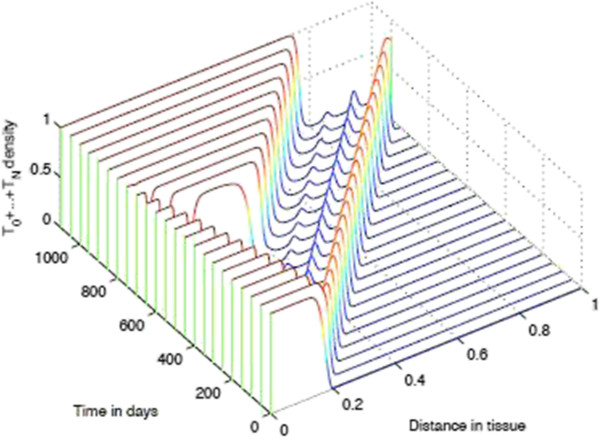
**Changes in the spatial distribution of all tumour cells**$\left(\overset{N}{\underset{j=0}{\sum }}T_{j}\right)$**within the tissue for decreasing*p***_***i***_**and costant**$\left(k_{i}^{+}\right)$**.** Plots showing detailed changes in the spatial distribution of all tumour cells $\overset{N}{\underset{j=0}{\sum }}T_{j}$ within the tissue over time in the case of immunoevasion. Parameter values *p*_*N*_=0.5 and $\left(k_{i}^{+}=\text{constant}\right)$.

**Figure 19:**
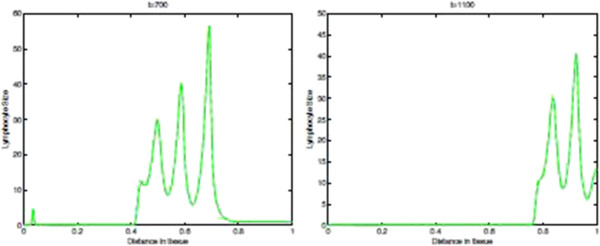
**Distribution of CTLs within the tissue for decreasing*p***_***i***_**with*p***_***N***_**=0.5**, and costant $\left(k_{i}^{+}\right)$**.** Plots showing the distribution of CTLs within the tissue at times corresponding to 700, and 1100 days respectively. These plots illustrate the spatiotemporal onset of immunoevasion. Parameter values *p*_*N*_=0.5 and $\left(k_{i}^{+}\right)$ are constant. Solid line with chemorepellent, dashed line without.

Figure 17 shows the distribution of tumour cell density within the tissue at times corresponding to 700, and 1100 days respectively with parameter values *p*_*N*_=0.5 and $\left(k_{i}^{+}=\text{constant}=k_{0}^{+}\right)$. Note that in this case, although *p*_*N*_=0.5, probably due to the constancy of $\left(k_{i}^{+}\right)$, in large parts of the space the number of naive cells exceeds the rest of the classes of other tumour cells, i.e. *T*_0_>*T*_1_ + ⋯ + *T*_*N*_. Note that at the end of the average lifespan of the mouse, the tissue is invaded to a large extent but to a lesser extent than in the case where *p*_*N*_=0.5 and $\left(k_{N}^{+}=0\right)$. Figure 18 shows a more detailed evolution of the tumour cell density by presenting the “time-slices” from *t*=0 to *t*=1100. Figure 19 shows the corresponding distribution of CTL density.

**Figure 20:**
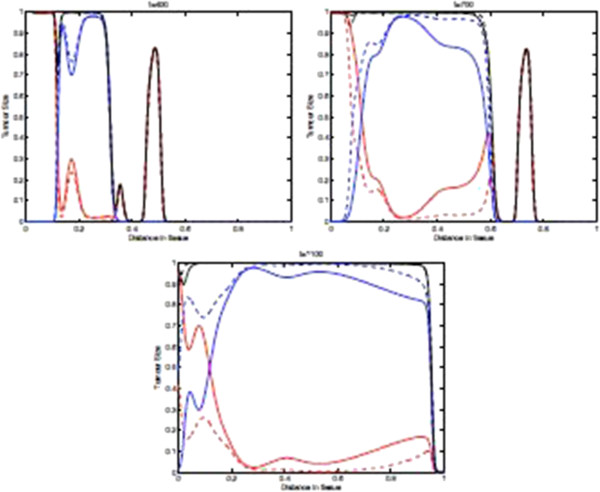
**Distribution of tumour cell density within the tissue for decreasing *p***_***i ***_**and **$\left(k_{i}^{+}\right)$, with ***p***_***N***_**=0.5 **and $\left(k_{N}^{+}=0\right)$**.** Plots showing the distribution of tumour cell density within the tissue at times corresponding to 400, 700, and 1100 days respectively. These plots illustrate the spatiotemporal onset of immunoevasion. Parameter values *p*_*N*_=0.5 and $\left(k_{N}^{+}=0\right)$. Solid line with chemorepellent, dashed line without. The red lines represent the population *T*_0_, The blue lines represent the summed population *T*_1_ + … + *T*_*N*_, and the black lines represent the summed population *T*_0_ + … + *T*_*N*_.

Finally, Figure 20 shows the distribution of tumour cell density within the tissue at times corresponding to 400, 700, and 1100 days respectively when the parameters *p*_*N*_=0.5 and $\left(k_{N}^{+}=0\right)$. This figure summarizes well the important role of the two parameters *p*_*N*_ and $\left(k_{N}^{+}\right)$ in shaping the spatio-temporal distribution of tumour cells. Indeed, the parameter $\left(k_{N}^{+}\right)$ appears to accelerate the onset and the velocity of propagation of the invasive front, and moreover it also differentially shapes *T*_0_ and *T*_1_ + ⋯ + *T*_*N*_.

## Conclusions

In this paper we have presented a novel mathematical model of the immune response to cancer, focusing on the specific spatio-temporal response of cytotoxic T-lymphocytes to tumour cells. We have developed and extended ideas originally formulated by [13] by proposing a possible kinetic mechanism leading to tumour evasion from the immune control. Our model is based on the key concept that a tumour cell which survives the formation of a complex with a cytotoxic T-lymphocyte can develop, with a given probability, an increased probability of surviving further attacks by CTLs. We do not specify whether this so-called ‘increased resistance’ is genetic or epigenetic. Indeed, from a kinetic point of view, this is immaterial. However, in order to experimentally validate our hypothesis, this distinction would be of paramount relevance.

Note that the model by [13] is based on a mass-action law mechanism. The reaction kinetics in a crowded cellular environment could differ from that, as studied (in absence of immunoevasion) in [17,27,28]. We shall investigate these more realistic settings in future works, but we think that the basic results showed here should not substantially change.

In this work we have dealt with the spatio-temporal interplay between tumours and a specific immune response from CTLs. We chose this approach because of the experimental evidence on the relevance of CTLs in determining tumour dormancy or the evasion of many important tumours such as melanomas, ovarian carcinomas and colorectal carcinomas, where the presence of infiltrating lymphocytes is a useful prognostic marker [42,46]. However, tumour immunoevasion from dormancy is a multi-faceted phenomenon. We stress here that by no means do we think that ours is an exhaustive theoretical treatment of a such complex phenomenon.

Concerning spatial issues we also briefly mention that also in case of small dormant tumours the next generation of spatial models of tumour growth should better stress and investigate the interplay of tissue 3D geometry and the tumour vascularization with phenotypic changes in tumour cells. We have built our model based on the tumour dormancy mathematical model of [13,16], where parameters were fitted to experimental animal (mouse) data. However, embedding the proposed evolutionary mechanism in a more complex setting, where a more detailed description of both adaptive and innate immunity is included, should lead to results qualitatively similar to those here illustrated.

Our simulations suggest that the proposed mechanism is able to mimic various dynamics of immunoevasion during the lifespan of a mouse. We have also highlighted the differential spatiotemporal contributions to evasion due, respectively, to: *i)* a decrease in the probability *p*_*i*_ of being lethally hit; *ii)* a decrease in the probability, embedded in $\left(k_{i}^{+}\right)$, that a tumour cell is recognized by a CTL. In particular, our model suggests that a decrease in the parameters *p*_*i*_is needed to produce evasion, which does not occur in the case where *p*_*i*_ remains constant at its baseline level inferred from the experimental data. However, the role of the parameters $\left(k_{i}^{+}\right)$ is important since it can greatly accelerate the simulated process. Moreover, our computational simulations also showed that the proposed mechanism can also deeply affect the spatial patterning of the tumour. In particular, our model suggests that to have a uniform invasion profile for the tumour cells necessitates also having a decrease in the recognition rate, embedded in the parameters $\left(k_{i}^{+}\right)$. These parameters also differentially shape the spatial distribution of the various classes of tumour cells.

Concerning the possible chemorepulsion of CTLs, our computational simulation results showed that, in our biological settings, although it does not affect the spatiotemporal dynamics of the total number of tumour cells, it has a remarkable influence on the spatio-temporal distribution of the different individual classes of tumour cells. Further analysis is needed to ascertain if, with different parameters, the effect of this factor can be different, and in order to understand the behaviour in the current setting.

As far as the key ‘immuno-evasion-related’ parameters such as *θ*_*i*_, *p*_*i*_, and $\left(k_{i}^{+}\right)$ are concerned, we were not able to fit them with experimental data (apart, of course, from the values for *p*_0_ and $\left(k_{0}^{+}\right)$, from [13,16]) because in the literature, to the best of our knowledge, immuno-evasion of tumours is only illustrated by means of qualitative clinical or molecular experimental findings. In particular, no immuno-evasion-related tumour growth data are available. Indeed, a complete experimental kinetic study of the adaptive evasion from tumour dormancy allowing, for example, the plotting of tumour growth curves would currently be very difficult to undertake. Thus we hope that this theoretical work may contribute to triggering such experimental investigations, which would allow us to validate our model.

From a theoretical point of view, our model, although detailed and focused on a very specific aspect of immuno-oncology, and on some very specific mechanisms, is conceptually in line with the general theories by Bellomo [36,37,41], who considers tumour cells and immune system effector cells as “active particles” endowed with activities and properties. Indeed, also in this paper the changes of activities of cells upon encounters between tumour cells and effector cells of the immune system are central in determining the dynamics of the system.

To the best of our knowledge, the evolutionary nature of the immuno-editing process has been studied until now under the framework of the so-called “modern synthesis”, following which the environment (in our case the immune system) is not the “causative agency” [47] but a mere selective force promoting fixation of adaptive genomic changes [47]. In the case of immunoevasion, a lowly immunogenic clone may appear spontaneously due to the large random mutation rate of tumour cells. This new phenotype is then involuntarily selected by the immune system, which kills the other phenotypes that remain strongly immunogenic. Thus the sculpting of tumour cells phenotypes [9] mentioned in the introduction, is involuntary, passive.

On the contrary, in our model the more immuno-resistant phenotypes may arise because of genetic or epigenetic causes - due to the interaction between the tumour cells and the immune system. This point of view, which is quasi-Larmackian [47], is in line with a number of recent discoveries that are leading to a new theory of “extended evolutionary synthesis” [48], which indeed investigates the impact of both genetic and epigenetic inheritance on evolutionary phenomena in order to decipher the complex interplay between genotypes, epigenotypes, phenotypes and environment. From a biological and biophysical point of view this implies also including two other key components: timescales (see, e.g., the review paper [49]) and the role of randomness. Following this extended and more modern perspective, we think that two general classes of evolutionary mechanisms might biologically underlie our kinetic model of transitions between phenotypes of tumour cells: stress-induced mutations and epigenetic switches.

In stress-induced mutagenesis [47,49], some kind of stress induces genomic changes in a cell that, although probabilistic (e.g. in our model *θ*_*i*_∈(0,1)), are triggered by the cell and its interplay with the “external world”, and that are beneficial for the cell [47,49]. As a result, the stress-induced mutations are adaptive and beneficial, and thus they are natural candidates to explain biologically the kinetic mechanism of our mathematical model. Moreover, it is important to note that stress-induced genomic changes are a well-known and important mechanism in tumours [47,50,51]. Finally, Koonin hypothesized [47] that quasi-Larmarckian processes are essentially triggered by very strong signals (see e.g. Figure 3 of [47]), which, we remark, is the case for tumour-CTL interplays.

As far as the epigenetic path is concerned, it is now known that there are inheritable phenotypic changes without underlying genetic variations [48,49], and that sometimes those changes are unusually rapid [49]. The “epigenome” is dynamic and it reflects an individual’s or a tissue’s environmental exposure during the whole of its lifespan [52]. Among the possible epigenetic changes, we mention specifically the methylation of DNA bases (“epimutations”), and also the switching between two different equilibrium states in the biochemical pathways influencing a set of inter-related phenotypes (e.g. “low immunogenicity” and “large immunogenicity”), due for example, to strong stress signals. Indeed, due to hysteresis phenomena inducing memory, also when an external stress signal is removed the system may not return to its original state. This memory may remain stable for many generations thus providing an example of epigenetic inheritance [49].

In this work we were interested essentially in the basic facts of the immune response to tumours. However, a number of immunotherapies have been proposed and also theoretically investigated (see [17,26,30] and references therein). We believe that both the experimental results concerning immunoevasion of tumours and the theoretical findings we have proposed here might have some implication of interest for clinical practice. Indeed, the immunoevasion mechanisms may be seen as a process counter-acting immunotherapies able to maintain a tumour in a dormant state. In general we share the opinion of [46], who stressed that recent progress in immuno-oncology has not influenced the way anti-cancer therapies are conceived and applied clinically. Indeed, we think that the immuno-editing process can be seen as an “involuntary” antagonistic process acting against immunotherapies. As far as this point is concerned, in future we will also investigate the possibility that the presence of an immunotherapy is somewhat sensed by a tumour, with a consequential acceleration of immuno-editing.

The model that was proposed here has to be understood as a detailed model at the level of the kinetics of the cellular populations of a possible mechanism that might enable tumour cells to evade from the control of adaptive immunity. The various specific (and tumour-dependent) strategies deployed by those cells in order to reach their aim, are phenomenologically described by means of the model of the dependence of the various parameters on the classes of tumour cells, as well as in the macroscopic modelling of the chemorepulsion. This is a first step in a research effort for a more complete description of tumour cell immunoevasion, which will include the detailed modelling of the biological mechanisms underlying those and other specific evasion strategies. Thus, given the complex network of interplaying between inter-cellular and intra-cellular signalling, and given the various temporal scales (from the rapid dynamics of the intracellular pathways involved, to the relatively slow growth of a tumour, up to the very slow onset of immunoevasion) as well as the spatial ones (from individual cells to visible tumours), a more detailed model will have to be multiscale. This will involve a wide array of computational tools, from those typical of computational biology and bioinformatics, to more classical analytical and numerical methods of statistical mechanics and mathematical physics.

## Appendix

### Non-dimensionalization

Before undertaking any computational simulations, we non-dimensionalise our model (as well as the boundary and initial conditions) by adopting the following scaling: *i)* space is scaled by adopting a reference value *x*_*a*_ equal to the size of the domain in consideration and we assume *x*_*a*_=1*cm*; time is rescaled relative to the diffusion rate of CTLs by setting $\left(t_{a}=x_{a}^{2}D_{E}\right)$:

(20) $$\bar{x}=\frac{x}{x_{a}},\;\;\;\bar{t}=\frac{t}{t_{a}};$$

*ii)* cellular densities are rescaled relative to the maxima of the respective initial conditions:

(21) $$\begin{array}{lll} & \;\;\;{\bar{T}}_{i}=\frac{T_{i}}{T_{a}},\;\;\;i=0,\ldots ,N\; & \;\\ & Ē=\frac{E}{E_{a}},\;\;\;{\bar{C}}_{l}=\frac{C_{l}}{C_{a}},\;\;\;l=0,\ldots ,N,\; & \; \end{array}$$

where, we recall, *E*_*a*_=*C*_*a*_; *iii)* The concentrations of the chemoattractant and of the chemorepellent are rescaled relative to the baseline values *α*_*a*_ and *ρ*_*a*_respectively:

(22) $$\bar{\alpha }=\frac{\alpha }{\alpha_{a}},\;\;\;\bar{\rho }=\frac{\rho }{\rho_{a}}$$

By omitting the bars, for the sake of simplifying the notation, the proposed model becomes:

(23) $$\begin{array}{lll} \frac{\mathrm{\partial E}}{\mathrm{\partial t}}\;\; & =\;\;\nabla^{2}E-\gamma (\alpha )\nabla (E\nabla \alpha )+\xi (\rho )\nabla (E\nabla \rho )\; & \;\\ & \;\;\;+\eta_{1}\frac{\overset{N}{\underset{j=0}{\sum }}q_{j}C_{j}}{a+\overset{N}{\underset{j=0}{\sum }}T_{j}}-E\left(\overset{N}{\underset{j=0}{\sum }}\psi_{j}T_{j}\right)-\mathrm{\sigma E}\; & \;\\ & \;\;\;+k_{3}\left(\overset{N}{\underset{j=0}{\sum }}C_{j}\right)+k_{4}\left(\overset{N}{\underset{j=0}{\sum }}p_{j}C_{j}\right)+\mathrm{\sigma h}(x),\; & \;\\ \frac{\partial \alpha }{\mathrm{\partial t}}\;\; & =\;\;D_{\alpha }\nabla^{2}\alpha -\delta_{\alpha }\alpha +\mu_{\alpha }\left(\overset{N}{\underset{j=0}{\sum }}\Pi_{j}C_{j}\right),\; & \;\\ \frac{\partial \rho }{\mathrm{\partial t}}\;\; & =\;\;D_{\rho }\nabla^{2}\rho -\delta_{\rho }\rho +\mu_{\rho }\left(\overset{N}{\underset{j=0}{\sum }}w_{j}T_{j}\right),\;\\ \frac{\partial T_{0}}{\mathrm{\partial t}}\;\; & =\;\;\nabla^{2}T_{0}+rT_{0}\left(1-\overset{N}{\underset{j=0}{\sum }}T_{j}\right)\; & \;\\ & \;\;\;-\phi_{0}ET_{0}+k_{1}C_{0}+(1-\theta_{0})(1-p_{0})k_{2}C_{0},\; & \;\\ \frac{\partial T_{i}}{\mathrm{\partial t}}\;\; & =\;\;\nabla^{2}T_{i}+rT_{i}\left(1-\overset{N}{\underset{j=0}{\sum }}T_{j}\right)-\phi_{i}ET_{i}+k_{1}C_{i}\; & \;\\ & \;\;\;+\theta_{i-1}(1-p_{i-1})k_{2}C_{i-1}\; & \;\\ & \;\;\;+k_{2}(1-\theta_{i})(1-p_{i})C_{i},\; & \;\\ \frac{\partial C_{l}}{\mathrm{\partial t}}\;\; & =\;\;\psi_{l}ET_{l}-\lambda C_{l},\; & \; \end{array}$$

where:

(24) $$\;\begin{array}{llll} r=r_{1}t_{a} & \phi_{i}=k_{i}^{+}E_{a}t_{a} & k_{1}=\frac{k^{-}C_{a}t_{a}}{T_{a}} & a=\frac{g}{T_{a}}\\ \lambda =t_{a}(k^{-}+k) & \psi_{i}=k_{i}^{+}T_{a}t_{a} & k_{2}=\frac{kC_{a}t_{a}}{T_{a}} & \gamma =\alpha_{a}t_{a}\chi \\ \eta_{1}=\frac{ft_{a}}{T_{a}} & \sigma =\mu_{o}t_{a} & k_{3}=k^{-}t_{a} & \mu_{\alpha }=\frac{C_{a}t_{a}}{\alpha_{a}}\\ D_{\alpha }=D_{2}t_{a} & \delta_{\alpha }=\delta_{1}t_{a} & k_{4}=kt_{a} & \mu_{\rho }=\frac{T_{a}t_{a}}{\rho_{a}}\\ \xi =\alpha_{a}t_{a}A & D_{\rho }=D_{*}t_{a} & \delta_{\rho }=\delta_{2}t_{a} \end{array}$$

After the non-dimensionalization, the boundary conditions become (in 1-D):

(25) $$\begin{array}{lll} \frac{\mathrm{\partial E}}{\mathrm{\partial x}}(0,t) & =0,\;\;\;\frac{\mathrm{\partial E}}{\mathrm{\partial x}}(1,t)=0,\; & \;\\ \frac{\partial \alpha }{\mathrm{\partial x}}(0,t) & =0,\;\;\;\frac{\partial \alpha }{\mathrm{\partial x}}(1,t)=0,\; & \;\\ \frac{\partial \rho }{\mathrm{\partial x}}(0,t) & =0,\;\;\;\frac{\partial \rho }{\mathrm{\partial x}}(1,t)=0,\; & \;\\ \frac{\partial T_{i}}{\mathrm{\partial x}}(0,t) & =0,\;\;\;\frac{\partial T_{i}}{\mathrm{\partial x}}(1,t)=0,\; & \; \end{array}$$

which then imply, assuming some smoothness of the solution and the form of *C*_*i*_equations, that

(26) $$\begin{array}{ll} \frac{\partial C_{i}}{\mathrm{\partial x}}(0,t)=0,\;\;\;\frac{\partial C_{i}}{\mathrm{\partial x}}(1,t)=0 & \; \end{array}$$

and the initial conditions take the form (again in 1-D):

(27) $$\;\begin{array}{ll} E(x,0) & =\left\{\begin{array}{ll} 0, & \;\;\;\;\text{if}\;0\leq x\leq l\\ (1-\text{exp}(-1000{\left(x-l\right)}^{2})), & \;\;\;\;\text{if}\;l<x\leq 1 \end{array}\right)\\ & \;\;\;\alpha (x,0)=0,\forall x\in [0,1]\\ & \;\;\;\rho (x,0)=0,\forall x\in [0,1]\\ T_{0}(x,0) & =\left\{\begin{array}{ll} (1-\text{exp}(-1000{\left(x-l\right)}^{2})), & \;\;\;\;\text{if}\;0\leq x\leq l\\ 0, & \;\;\;\;\text{if}\;l<x\leq 1 \end{array}\right)\\ & \;\;\;T_{i}(x,0)=0,\forall x\in [0,1]\\ C_{0}(x,0) & =\left\{\begin{array}{ll} 0, & \;\;\;\;\;\text{if}\;x\notin [l-\varepsilon ,l+\varepsilon ]\\ \text{exp}(-1000{\left(x-l\right)}^{2}), & \;\;\;\;\;\text{if}\;x\in [l-\varepsilon ,l+\varepsilon ] \end{array}\right)\\ & \;\;\;C_{i}(x,0)=0,\forall x\in [0,1] \end{array}$$

where

(28) l=0.2,ε=0.01.

The chosen non-null initial conditions for the naive tumour and immune cells represent a front of tumour cells encountering a front of CTLs, resulting in the formation of CTL-tumour cell complexes. In the absence of a tumour, the homogeneous steady-state density of the CTLs is *s*/*d*_1_ and therefore this is the value we have taken for the initial density *E*_*a*_of CTLs in the initial conditions. Similarly, in the absence of an immune response, the homogeneous steady-state density of the tumour cells is 1/*β*_1_ and this is what we take as the initial density of tumour cells *T*_*a*_in the initial conditions. Thus, when the fronts of the two cell populations meet, the maximum density of CTL-tumour cell complexes will be *min*(*E*_*a*_,*T*_*a*_) and hence our choice for *C*_*a*_. The function exp(−1000(*x*−*l*)^2^) was chosen to mimick the onset of sharp but continuous front.

### Numerical values of the parameters

To carry out the computational simulations of the proposed model, we used the baseline parameter set reported in [13], since these were all estimated from experimental data on murine B-cell Lymphoma *BCL*_1_, which is an animal model for the study of tumour dormancy [53]. In addition to this baseline set, we used the migration parameters proposed in [16]. Thus, the complete set of parameters, and of their meaning, is the following (TC stands for tumour Cell):

(29) $$\;\begin{array}{ll} s=1.36\times 10^{4}\;\text{da}y^{-1}\text{cells}\;cm^{-1}\;\;\; & \text{CTL supply rate}\\ f=0.2988\times 10^{8}\;\text{da}y^{-1}\text{cells}\;cm^{-1}\;\;\; & \text{Complex-induced}\\ \text{proliferatiomn rate}\\ \text{constant}\\ g=2.02\times 10^{7}\;\text{cells}\;cm^{-1}\;\;\; & \text{Constant appearing}\\ \text{in proliferation function}\\ k_{0}^{+}=1.3\times 10^{-7}\;\text{da}y^{-1}\text{cell}s^{-1}\;\text{cm}\;\;\; & \text{‘Formation rate’ of}\\ \text{complexes}\\ k^{-}=24\;\text{da}y^{-1}\;\;\; & \text{Complexes ’unbinding}\\ \text{rate’}\\ k=7.2\;\text{da}y^{-1}\;\;\; & \text{Rate of lethally hit TC or}\\ \text{CTL rate}\\ p_{0}=0.9997\;\;\; & \text{Death probability of}\\ \text{naive TC}\\ r_{1}=0.18\;\text{da}y^{-1}\;\;\; & \text{Baseline exponentil}\\ \text{growht rate of TCs}\\ \beta_{1}=2\times 10^{-9}\;\text{cell}s^{-1}\;\text{cm}\;\;\; & \text{Inverse of tumour}\\ \text{carrying capacity}\\ d_{1}=0.0412\;\text{da}y^{-1}\;\;\; & \text{Baseline death rate}\\ \text{of CTLs}\\ D_{E}=10^{-6}\;cm^{2}\;\text{da}y^{-1}\;\;\; & \text{Diffusion coefficient}\\ \text{of CTLs}\\ \chi =1.728\times 10^{6}\;cm^{2}\;\text{da}y^{-1}\;M^{-1}\;\;\; & \text{Chemotaxis coefficient}\\ \text{of CTLs}\\ D_{T_{i}}=10^{-6}\;cm^{2}\;\text{da}y^{-1}\;\;\; & \text{Diffusion coefficient}\\ \text{of TCs}\\ D_{2}=8\times 10^{-3}cm^{2}\;\text{da}y^{-1}\;\;\; & \text{Diffusion coefficient}\\ \text{of chemorattractant}\\ \delta_{1}=1.15510^{-2}\mathrm{day}s^{-1}\;\;\; & \text{degradation rate of the}\\ \text{chemoattractant} \end{array}$$

Hence, from the experimental data above, the non-dimensional values of parameters becomes:

(30) $$\;\begin{array}{lll} \gamma =1.728\times 10^{2} & \;\;\;\eta_{1}=5.976\times 10^{4} & \;\;\;\psi_{0}=6.5\times 10^{7}\\ \sigma =4.12\times 10^{4} & \;\;\;k_{3}=2.4\times 10^{7} & \;\;\;k_{4}=7.2\times 10^{6}\\ D_{\alpha }=8\times 10^{3} & \;\;\;\delta_{\alpha }=1.115\times 10^{4} & \;\;\;\mu_{\alpha }\Pi_{0}=10^{4}\\ r=1.8\times 10^{5} & \;\;\;\phi_{0}=4.29\times 10^{4} & \;\;\;k_{1}=1.584\times 10^{4}\\ k_{2}=4.752\times 10^{3} & \;\;\;\lambda =3.12\times 10^{7} & \;\;\;a=4.04\times 10^{-2} \end{array}$$

As far as the spatiotemporal dynamics of the chemorepellent is concerned, we assume that its diffusion coefficient and decay rate is the same as the chemokine *α*i.e. *D*_*ρ*_=*D*_*α*_, *δ*_*ρ*_=*δ*_*α*_, *μ*_*ρ*_=*μ*_*α*_, and *ξ*=*γ*.

Note that in the first table above we did not provide the value of *Π*_0_, wherease in the second table we directly provided the adimensional value *μ*_*α*_*Π*_0_=10^4^, in line with [13,16].

## Reviewers’ comments

The comments of the referees were reported in italics. All the three referees included minor comments on misprints or undefined parameters or other minor suggestions, which were all implemented. Thus, we only reported the comments of interest to the general readership.

Note for the referees: following your suggestions the revised version now contains an Appendix.

### Reviewer #1: Prof. G. Bocharov (nominated by Dr. V. Kuznetsov, member of the Editorial Board of *Biology Direct*) (Institute of Numerical Mathematics, Russian Academy of Sciences, Moscow, Russian Federation)

The paper presents a theoretical study of the tumour immuno-evasion from dormancy. To this end a mathematical model of reaction-diffusion-chemotaxis type formulated with PDE is proposed. The model considers the spatio-temporal population dynamics of interactions between tumour cells and cytotoxic T cells. The key assumption of the model reflecting recent biological insights into the pathogenesis of the solid tumour growth states that the tumour cell population is heterogeneous with respect to the parameters characterizing the outcome of the interaction with cytotoxic T lymphocytes. The interaction with CTL appears to acts as a selection force shaping the microevolution of the tumour. The heterogeneity assumption enters the model via parameterized dependence of the rate of transition of tumour cells from naive to more mature states, the efficacy of CTL mediated killing and the production of chemicals acting as attractants and repellors for CTLs. Numerical simulations with the model for various parameters combinations show that the immuno-evasion can result from the phenotypic heterogeneity of the tumour cells dynamically adapting to and shaped by the anti-tumour immune response.

General remarks:

1. The issue of spatio-temporal modelling of tumour growth is an area of active research in cell population dynamics (e.g., the studies of Bertuzzi and Gandolfi). The related work on spatio-temporal modelling of tumour growth needs to be refereed to in the Introduction section.

We fully agree, and we added the required references.

Page 6: The authors make use of the Mass Action Law to describe the interaction between CTL and target cells via bilinear terms. Meantime, the reaction kinetics in crowded cell environments can differ from those assumed by the classical chemical kinetics. This issue need to be commented.

This point is very important, and we commented it in the conclusions.

*Page 10: The parameter “1000” appearing in the initial functions has to be justified. Please, explain the choice ofC*_*a*_**.**

We added a full justification of the chosen initial conditions.

Page 13: For the readership, the parameters of the model should be presented in a Table with columns specifying notation, biological meaning, units and uncertainty ranges.

We added a table with average values of the parameters, as obtained in Ref. 13 by means of least squares algorithm, and their meaning.

Page 13, line 20: Can the parameters equality assumption for the repellor and attractant be biologically justified?

Unfortunately there is a lack of experimental results on this important data, so this is a pure assumption.

Page 14, line -5: Please, elaborate more on the specific choice of the parameter values.

We added a reference for this parameter. More in general all parameters in absence of immuno-editing were taken as in references [13,16].

*Page 15-16: Figures*3-8*could be presented in a more compact way (e.g., as array of graphs). The same applies to Figures*9-19.

We tried your suggestion, but the result was not less clear. Take also into the account that Biology Direct is purely online, thus there are no pages restrictions.

Page 18: Section 2-Dimensional Domain is based upon simulations with the chemotaxis and chemorepulsion not included in the model. The value of the results of the 2D model based simulations is not straightforward. Please, either expand the section by considering the same set of processes as in 1D case or remove it.

We removed it.

Conclusions section: It would be interesting to have the authors opinion on whether the tissue 3D geometry and its vascularisation represent important structural constraints affecting the genomic or phenotypic changes in tumour cells that need to be considered in the next generation of the spatially resolved models of tumour growth?

We fully agree, and we mentioned this important issue in the discussions.

Finally, all suggestions in your minor remarks were implemented.

### Reviewer #2: Prof. M. Kimmel (Rice University, Houston, USA)

The paper concerns an extension of Chaplain group’s model (Matzavinos et al. 2004) of response of tumour cells to cytotoxic T-lymphocytes. The extension involves classes from naive through non-naive tumour cells, which have different ability to repel T-lymphocytes and other differing characteristics. The models consists of a complicated system of partial differential equations and is investigated purely by simulation.

In this first investigation we employed numerical analysis for two main reasons: first, we feel the immuno-editing phenomena as mainly a transitory effect, and as such requiring mainly numerical analysis; second, we wanted to write a manuscript aimed to theoretical biologists with biological background, such as the general readership of ’Biology Direct’. However, we also outlined a more compact version of the proposed model where the phenotypes are described by means of a continuum. For this version we soon start a full mathematical analysis.

In my opinion, stratification of tumour cells with respect to their immuno-naivete is a natural concept, which however, leads to many complications and ambiguities in model building. The paper only partially reviews the consequences of varying the hypotheses.

In the above mentioned continuum version we should be able of better exploring these important issues.

In particular, the 2-d model by the end of the paper seems superfluous, as it is not thoroughly investigated and seems to be plagued by boundary artifacts. I suggest removing it for the time being.

Following you suggestions, and the suggestions of another referee, we removed it.

I am not sure if the tumour immuno-editing can be considered a Lamarckian evolutionary process. Even if it is, I am not sure if this is important for the paper.

We respectfully disagree with you on this point. The possibility that immuno-editing might be, at least in some cases, a Lamarckian process, where the interplay between the different kind of cells is the ’driver’, is of some interest, in our opinion.

Page 2. I do not know if “extremely complex” implies “strongly non-linear” as the authors seem to claim.

We removed this observation.

Page 2.(bottom). It would be perhaps helpful to specify which cell types belong to the adaptive immune system and which to the innate one.

Done.

Page 5. “Since we are modeling a situation where immuno-evasion of the tumour cells is not considered …” Discussion of what this assumption really implies will be helpful.

We removed the above quite ambiguous phrase. Indeed, we wanted to means that the baseline model, with the considered values of its parameters, referred to a case where the tumour is not able (without immuno-editing) to evade the immune control.

Pages 9-13. Part of this material can probably go into an online Appendix.

Done.

### Reviewer #3: Prof. A. Marciniak-Czochra (Heidelberg University)

The paper is devoted to modelling of spatio-temporal dynamics of interactions of cytotoxic T-lymphocytes with cells of a homogeneous avascular tumour. It takes into consideration a chemoattraction of the immune cells by the tumour and a chemorepulsion to chemicals produced by non-naive tumour cells. The model is based on the one presented in the paper “Mathematical Modelling of the spatio-temporal Response of Cytotoxic T-Lymphocyte to a Solid Tumour” (Matzavinos A, Chaplain M, Kuznetsov, Mathematical Medicine and Biology, 2004, 21:1-34) The novelty of the current work is in the assumption that the tumour cells, surviving encounter with the immune cells, undergo a functional change, which benefits their immune resistance. This resistance increases with each encounter until the maximal resistance is reached. The resulting system of coupled partial and ordinary di erential equations is solved numerically. The results are discussed in the biological context. I recommend the manuscript for publication in Biology Direct after some minor revisions related to the organisation of the paper. The issues, which should be taken into account before publication are (Note of authors:this referee listed a long series of misprints, which were all fixed. We only report the comments of interest for the reader):

P.8, the second Equation: f and g are not introduced. Although model equations are nearly identical to those in reference (Matzavinos et al, 2004), authors should motivate the term “proliferation and recruitment”.

In the revision, we fully explained the meaning of the term like *fC*/(*g* + *T*).

P.10: It would be helpful to have the initial conditions plotted.

Since the shape of the initial conditions are elementry, and since another referee asked to add some written comemnts on this point, we did not include the requested plots.

P.13: It should be mentioned how parameters are estimated.

The parameters were estimanted in Kuznetsov *et al* 1991 basically by means of mininmal least squares estimate algorithm.

*Please motivate the choice of θ*_0_; *p*_*N*_; $\left(k_{0}^{+}\right)$; *how sensitive are the results to the choice of the parameters?*

We have choosen a small value for *θ*_0_because we think that the probability of acquiring the fitter phenotype is small, in analogy with the small probability of surviving to an attack by a CTL. The value of $\left(k_{0}^{+}\right)$ is taken from (Matzavinos *et al* 2004).

*P. 28, Figure *12: *Why (700,1100) and not (800, 1100)?*

800 days would have been a too long time with respect to 700 (100 days in mice correspond approximatively to 2300 days in men).

Axis labels of the following figures are not readable: 9, 10, 11, 13, 15, 17, 20, 21.

Concerning the readability of the axis labels, it probably depend on the fact that our eps figures were converted by the publisher in jpeg figures. In our original pdf figures they seems readable. We shall carefully manage this point in case the manuscript should be accepted.

### *The following were comments on the revised manuscript (other comments on missprints are omitted, and were implemented):*

*The rescaling of equations have mistakes. For example on p. 30*, *λshould be*$\left(t_{a}(k^{-}+k)\right)$,

Indeed, it was a misprint. We apologize.

*while*$\left(\psi_{i}=k_{i}^{+}E_{a}T_{a}t_{a}/C_{i}\right)$.

We had forgot to stress that *C*_*a*_=*E*_*a*_, so what we wrote is correct.

I find the following phrase hardly readable “Finally, we note that the predicted effect on the tumour cells spatial distribution of the induction of chemorepulsion of CTLs is not detectable if we consider the total density of all tumour cells.” Please refomulate it.

We reformulated it.

## Competing interest

The authors declare that they have no competing interests.

## Author’s contributions

AD and MC defined the mathematical model; MAT performed the simulations; AD, MC and MAT discussed the results; AD,MC and MAT wrote the manuscript. All authors read and approved the final manuscript.
